# Challenges and Modification Strategies of Ni-Rich Cathode Materials Operating at High-Voltage

**DOI:** 10.3390/nano12111888

**Published:** 2022-05-31

**Authors:** Caijian Liao, Fangkun Li, Jun Liu

**Affiliations:** Guangdong Provincial Key Laboratory of Advanced Energy Storage Materials, School of Materials Science and Engineering, South China University of Technology, Guangzhou 510641, China; 202120121261@mail.scut.edu.cn (C.L.); 202110184208@mail.scut.edu.cn (F.L.)

**Keywords:** Ni-rich cathode materials, high voltage, surface degradation, microcrack, modification

## Abstract

Ni-rich cathode materials have become promising candidates for lithium-based automotive batteries due to the obvious advantage of electrochemical performance. Increasing the operating voltage is an effective means to obtain a higher specific capacity, which also helps to achieve the goal of high energy density (capacity × voltage) of power lithium-ion batteries (LIBs). However, under high operating voltage, surface degradation will occur between Ni-rich cathode materials and the electrolytes, forming a solid interface film with high resistance, releasing O_2_, CO_2_ and other gases. Ni-rich cathode materials have serious cation mixing, resulting in an adverse phase transition. In addition, the high working voltage will cause microcracks, leading to contact failure and repeated surface reactions. In order to solve the above problems, researchers have proposed many modification methods to deal with the decline of electrochemical performance for Ni-rich cathode materials under high voltage such as element doping, surface coating, single-crystal fabrication, structural design and multifunctional electrolyte additives. This review mainly introduces the challenges and modification strategies for Ni-rich cathode materials under high voltage operation. The future application and development trend of Ni-rich cathode materials for high specific energy LIBs are projected.

## 1. Introduction

Since the commercialization of lithium-ion batteries (LIBs) by Sony in 1991, they have received widespread attention and become the dominant energy storage devices in portable electronics and electric vehicles (EVs) [1,2,3,4]. Nowadays, EVs suffer from the anxiety of endurance mileage due to the limitation of energy density in power LIBs. Additionally, the spontaneous combustion of EVs has also attracted much attention. Therefore, improving the energy density and safety property of power LIBs is the focus of academia and industry [5,6,7,8,9].

In order to meet the requirements for high energy density (capacity×voltage) and high safety, it is of great significance to design a reasonable battery material system. Output voltage of LIBs is decided by the potential difference between the anode and the cathode [1]. As one of the anode materials for LIBs, graphite has been successfully applied to the market to a certain extent [10], so the output voltage of the battery is mainly determined by the cathode materials. Furthermore, the cathode materials serve as the provider of lithium-ions, which greatly determines the capacity of LIBs. Nowadays, a great deal of research work has been done to develop cathode materials with superior specific capacities at high operating voltage [11].

At present, the cathode materials can be mainly divided as follows: (1) Lithium oxide compounds with layer structure LiMO_2_ (M = Ni, Co, Mn, Al), such as LiCoO_2_ [12] LiNi_x_Co_y_Mn_1−x−y_O_2_ (NCM) [13] and LiNi_x_Co_y_Al_1−x−y_O_2_ (NCA) [14] (also known as ternary cathode materials, TCMs); (2) Compounds with spinel structure, such as LiMn_2_O_4_ [15,16] and a series of Li_1+x_Mn_2−y_O_4_ spinels [17]; (3) Polyanionic structure compounds—the typical material with olivine-type structure is LiFePO_4_ [18]. Among the above cathode materials, Ni-rich cathode materials in TCMs with high capacity have become the principal candidates for long-range EVs [19,20]. With high nickel content, the cathodes can deliver the desired specific capacity. In addition, increasing the cut-off voltage can also improve the battery energy density. Unfortunately, when operating at high voltage, Ni-rich cathode materials will have challenges of rapid capacity attenuation and poor thermal stability [21,22]. The high working voltage will cause serious side reactions between the Ni-rich cathode materials and the electrolytes, resulting in the formation of a solid phase interface film, which shows high resistance on the cathode surface. Surface reconstruction occurs under the high voltage as well [23]. High voltage will accelerate the release of O_2_, CO_2_ and other gases, which greatly affects the safety performance of LIBs. Due to the high nickel content, the Ni-rich cathode materials have serious cation mixing, resulting in the transformation of the material structure from ordered layered phase to spinel and/or inert rock salt phase. In addition, the high working voltage will cause a large volume change, even microcracks, resulting in contact failure and repeated surface reactions.

**Figure 1:**
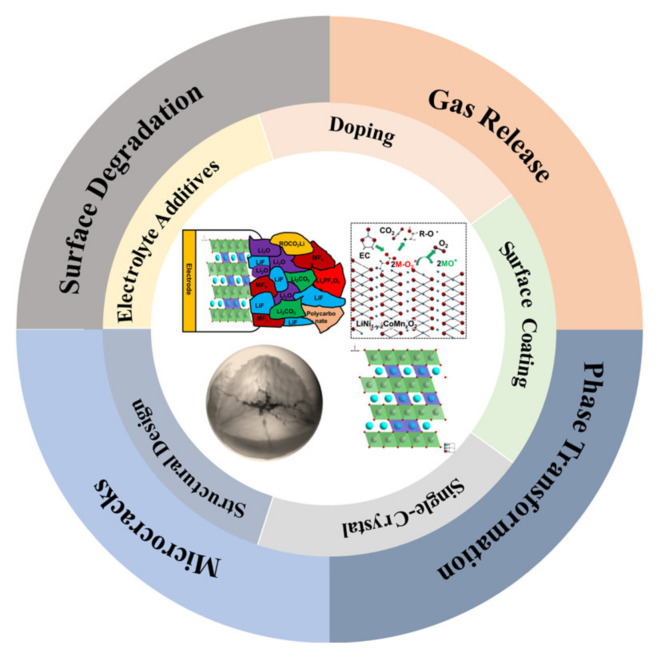
Challenges and modification strategies of Ni-rich cathode materials under high voltage.

In response to the above challenges for Ni-rich cathode materials under high voltage, researchers have proposed many modification methods to deal with the decline of electrochemical performance, such as element doping, surface coating, single-crystal fabrication, structural design and multifunctional electrolyte additives. This review mainly introduces the challenges and modification strategies for Ni-rich cathode materials under high voltage operation (Figure 1). The future application and development trend of Ni-rich cathode materials for high specific energy LIBs are projected.

## 2. Challenges of Ni-Rich Cathode Materials under High Voltage

Under high voltage operation, the layered Ni-rich cathode materials mainly face the following challenges: surface degradation, gas release, phase transformation and microcracks (Figure 1). Next, we will discuss these challenges under high voltage in detail.

### 2.1. Surface Degradation

The interface stability between Ni-rich cathode materials and other battery components is crucial for LIBs to obtain excellent electrochemical performance. The “electrochemical window” of the electrolyte, which is decided by the energy gap (*E*_g_) between the lowest unoccupied molecular orbital (LUMO) and the highest occupied molecular orbital (HOMO), limits the operating voltage of LIBs. The anode should be selected such that the electrochemical potential of the anode lies below the LUMO of the electrolyte, and the cathode must be picked so that the electrochemical potential of the cathode is located above the HOMO of the electrolyte; or else, the electrolyte will undergo reduction reaction at the anode or oxidation reaction at the cathode [24,25]. The passivated solid-electrolyte-interphase (SEI) and cathode-electrolyte-interphase (CEI) films might block charge transfer. Therefore, the electrolyte-electrode-interface plays a crucial role for the reversibility of lithium-ion intercalation and the kinetics of the battery reactions, eventually influencing the electrochemical performance of LIBs [26,27,28,29].

**Figure 2:**
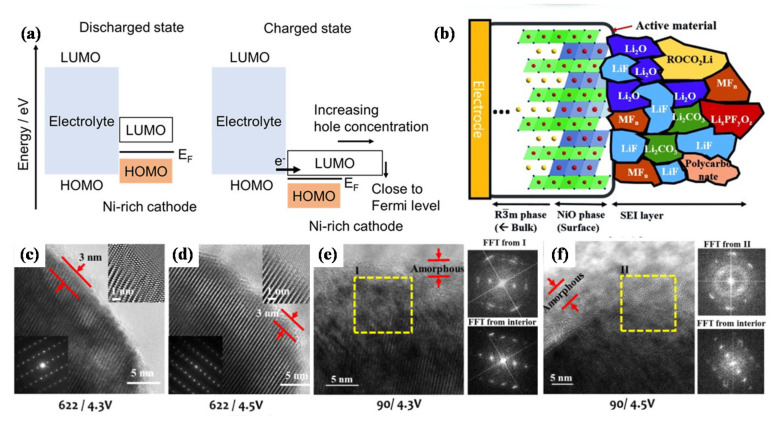
(**a**) LUMO and HOMO energy level diagrams of the Ni-rich cathode and electrolyte. Reproduced with permission from ref. [30]. Copyright 2020 American Chemical Society. (**b**) Schematic diagram of CEI film composition on Ni-rich cathode surface. Reproduced with permission from ref. [31]. Copyright 2015 John Wiley and Sons. TEM surface images and Fourier transform (after 100 cycles) for (**c**) NCM622 charged to 4.3 V, (**d**) NCM622 charged to 4.5 V, (**e**) NCM900505 charged to 4.3 V, (**f**) NCM900505 charged to 4.5 V. Reproduced with permission from ref. [32]. Copyright 2017 American Chemical Society.

The LUMO levels of transition metal ions in Ni-rich cathode materials are lower than that of other active materials, which will accelerate the oxidation of electrolytes. The energy for the transition metal (TM) 3d–O 2p antibonding hybrid orbital is equivalent to the LUMO energy level of the hole. As shown in Figure 2a [30], during the charging process, the Fermi level of the Ni-rich cathode is close to the HOMO level of the electrolyte. Additionally, the hole concentration in the charged state increases rapidly, resulting in the oxidation of the electrolyte. Figure 2b shows the CEI film formed on the surface of Ni-rich cathode [31], the inorganic components act as the “inner layer” of the CEI film and closely adhere to the electrode surface, the organic components are located at the outer layer of the CEI film, Manthiram et al. [32] also proved it by Time-of-flight secondary-ion mass spectrometry (ToF-SIMS). The CEI film is mainly composed of Li_2_CO_3_, alkyl carbonate and Li_x_PO_y_F_z_ oxidized from the electrolyte. Due to the presence of unstable Ni^4+^ with a high oxidation state on the surface of the cathode material, electrolyte oxidation occurs easily to form a thick CEI film. The lithium-ion conductivity of the CEI film is relatively poor, resulting in local resistance increases and performance degradation [31]. Thus, further research is necessary to form CEI films that possess high stability and ionic conductivity.

Surface reconstruction is another degradation mechanism for the interface between the cathode and electrolyte. During the phase transformation process, the original crystal structure transforms from a layered structure to a spinel-type structure, and then transforms into a NiO-like rock salt structure [33,34], forming a reconstruction layer on the surface of Ni-rich cathodes. The lithium migration kinetics of the formed rock salt structure is poor, resulting in the obstruction of lithium-ion diffusion channels and the decrease of ionic conductivity [35,36,37]. Doeff et al. [35] found that the impedance growth of NCM was closely associated with a surface structure under high-voltage operation and chemical evolution. The high-voltage cycling can increase the accumulation of rock salt layer on the surface, and the direct contact with electrolyte solution can also promote the formation of surface reconstruction layer, leading to the electrochemical performance degradation of the battery. In order to understand the capacity fading mechanism of Ni-rich cathode materials, Manthiram et al. [32] carried out a transmission electron microscopy (TEM) study on the surface of the cathode particles after cycling. At a voltage of 4.3 V, a thin layer of NiO-like structure is observed on the surface of LiNi_0.6_Co_0.2_Mn_0.2_O_2_ (NCM622). Increasing the cut-off voltage to 4.5 V has little effect on the surface structure of the NCM622 cathode. At a higher voltage of 4.5 V, the damaged surface layer is also limited to the thickness of 3 nm, and the layered structure is well retained inside the particles (Figure 2c,d). When the NCM900505 cathode is cycled at 4.3 V, the surface damage is obviously more extensive. As shown in Figure 2e,f, the thickness of the NiO-like layer increases to ~5 nm. Electrolyte oxidation occurs easily due to the large amount of thermodynamically unstable Ni^4+^ on the NCM900505 cathode surface, during the charging process, Ni^4+^ is reduced to Ni^2+^, while O^2−^ is oxidized to escape from the lattice to keep the charge neutral. The escape of a large amount of oxygen will generate a large number of oxygen vacancies, which will lower the activation energy barrier of transition metal cation migration so that the formation of rock salt structure is aggravated as well. Furthermore, the deformation of the damaged layer is serious, resulting in the formation of polycrystalline domains. There is also a thick amorphous layer material on the surface of NCM900505 cathode particles cycling at 4.5 V. The above analysis shows that at high voltage, the Ni-rich cathode materials with higher nickel content will occur more serious surface reconstruction. Grey et al. [38] proposed that when the electrode was charged to states of charge (SOC) above a threshold of around 75%, the lattice mismatch between the layered structure of NCM and the surface reconstruction layer of rock salt was the main reason for fatigue degradation. Their findings suggest the urgency of formulating effective modification strategies to enhance the performance of Ni-rich cathode materials.

### 2.2. Gas Release

**Figure 3:**
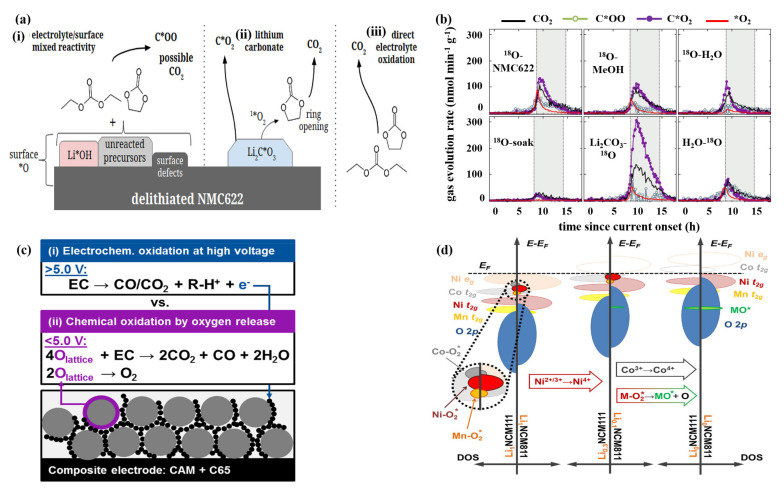
(**a**) The reasons for gas generation at the surface. “surface *O” indicates that the oxygen is from the surface of the NCM622 oxide or oxygen-containing impurities originally present on the surface (for example, Li*OH and Li_2_C*O_3_), and “O” indicates that the oxygen is from the electrolyte. (**b**) Rate of gas evolution for NCM622 with different treatments (^18^O–NCM622: the baseline sample synthesized via conventional procedure; ^18^O–MeOH: sample treated by methanol rinsing; ^18^O–H_2_O: sample treated by H_2_O rinsing; ^18^O–soak: sample treated by H_2_O soaking; Li_2_CO_3_–^18^O: sample treated by Li_2_CO_3_ enriching; H_2_O–^18^O: sample treated by H_2_O washing and ^18^O enriching). Reproduced with permission from ref. [39]. Copyright 2019 American Chemical Society. (**c**) Proposed gassing mechanisms in high-voltage lithium-ion cells. Reproduced with permission from ref. [40]. Copyright 2017 American Chemical Society. (**d**) Qualitative DOS diagrams for NCM111 and NCM811 at full lithiation, maximum nickel oxidation, and complete de-lithiation. “M-O_2_*” refers to the energy states of surface species. Reproduced with permission from ref. [23]. Copyright 2017 American Chemical Society.

The reaction of gas release in the battery is usually exothermic, which will cause a series of subsequent chain reactions, and ultimately lead to the occurrence of dangerous accidents. In Ni-rich cathode materials, there are three reasons for gas generation, as shown in Figure 3a [39]. The first reason, “surface *O” indicates that the oxygen is from the surface of the NCM622 oxide or oxygen-containing impurities originally present on the surface (for example, Li*OH and Li_2_C*O_3_), and “O” indicates that the oxygen is from the electrolyte. It has been considered that due to the existence of surface defects such as lithium vacancies, surface hydroxides and unreacted precursors, the mixed reactions between the electrode and electrolyte would generate carbon dioxide C*OO. Second, the decomposition of Li_2_C*O_3_ also leads to the increase of C*O_2_, and Li_2_C*O_3_ reacts with the electrolyte to release more carbon dioxide. Third, the direct electrochemical oxidation of the ethylene carbonate (EC)/diethyl carbonate (DEC) electrolyte also gives rise to CO_2_. Gasteiger et al. [40] have found that the lattice oxygen released from Ni-rich cathode materials reacts with the electrolyte to release CO_2_ and CO gases at a voltage less than 5 V. In this case, the oxidation rate of the electrolyte is determined by the surface area of the active electrode material and the conductive carbon. When the voltage is above 5 V, the electrolyte would undergo direct electrochemical oxidation reaction for gas evolution. Furthermore, the authors propose two mechanisms to explain electrolyte oxidation (Figure 3c): (1) Electrochemical oxidation of the electrolyte; and (2) Chemical oxidation of the electrolyte caused by the release of lattice oxygen. This work presents two unique oxidation mechanisms for the electrolyte, which also shows that further research is necessary to better understand the complexity of the gas production mechanism [40].

Under high SOC condition, the presence of surface defects will accelerate the generation of the gas. Washing is often used to alleviate side effects. McCloskey et al. [39] used different washing methods to study the effect of surface contamination on gas evolution in the de-lithiated NCM622 (Figure 3b). The NCM622 samples were prepared with different washing treatments (^18^O-NCM622, ^18^O-MeOH, ^18^O-H_2_O, ^18^O soak, Li_2_CO_3_-^18^O and H_2_O-^18^O) and kept at a high voltage of 4.8 V for a long time to measure release amount of the gas. The sample soaked in H_2_O does not give the best capacity because of the bulk de-lithiation of 0.82 Li, which is smaller than other samples. However, the results show that the gas evolution and irreversible bulk capacity loss of the sample soaked in H_2_O (^18^O-soak) are the smallest. In addition, the mechanism of gas formation affected by surface contaminants and defects is quite complex. Therefore, appropriate surface pretreatment is critical to reducing gas generation.

Besides, lattice oxygen losses caused by structural transition (LiMO_2_→MO or M_3_O_4_) give rise to the release of O_2_ as well [23]. Berg et al. [23] used the Online Electrochemical Mass Spectrometry (OEMS) to monitor the gas release of NCMs with different nickel contents. In the specific narrow range of the state of Ni oxidation (SNOX), CO_2_ and O_2_ begin to release in large quantities, and the gas release rate is significantly correlated with Ni content of NCMs. The electronic density of state (DOS) diagrams of NCM111 and NCM811 in three different states are shown in Figure 3d, where “M-O_2_*” refers to the energy states of surface species. Further, they have drawn a conclusion that the electron depletion rate of Ni-O_2_* surface state determines the formation rate of CO_2_ and O_2_ gases. The bulk oxidation of Co t_2g_ and surface states of Co–O_2_* have some influence on gas release to a lesser extent. From the correlation between CO_2_ and O_2_ gases release, it shows that surface reconstruction is the main contributor to electrolyte decomposition. These findings provide further understanding of the relationship between the composition and structural/surface stability of Ni-rich layered oxide cathode materials. It is significant to take rational modification measures for developing state-of-the-art cathode materials in LIBs and OEMS is a superior operando analytical instrument for guiding this development.

### 2.3. Failure Induced by Bulk Phase Transformation

**Figure 4:**
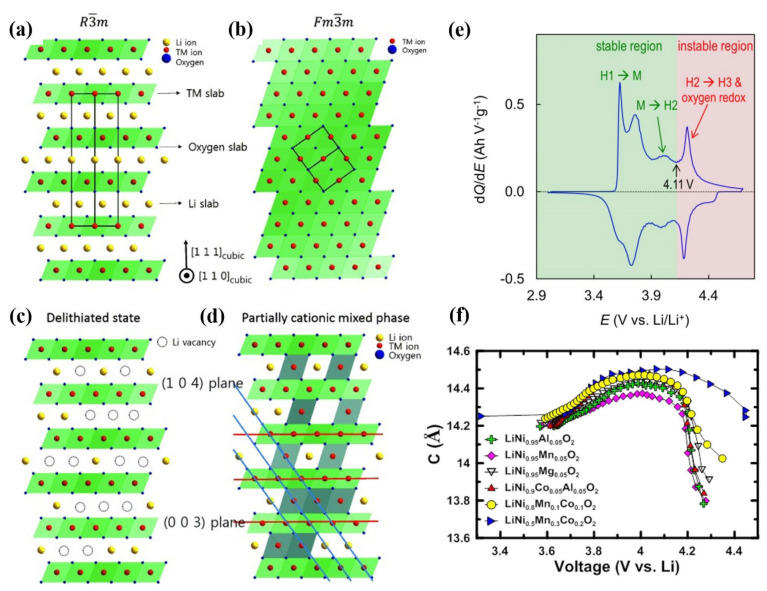
Schematic diagram of ordered and disordered phases and their structural transitions in layered lithium metal oxides. (**a**) Ordered R$\bar{3}$m structure. (**b**) Cation mixing phase with Fm$\bar{3}$ m structure. (**c**) R$\bar{3}$ m structure with Li vacancies at highly charged state. (**d**) Cation mixed phase with partial TM ions in Li layer. Reproduced with permission from ref. [31]. Copyright 2015 John Wiley and Sons. (**e**) Typical dQ/dV curves for Ni-rich NCM/Li cells. Reproduced with permission from ref. [46]. Copyright 2020 Elsevier. (**f**) c–axis lattice parameter as a function of x in Li_x_MO_2_. Reproduced with permission from ref. [50]. Copyright 2019 American Chemical Society.

LiMO_2_ (M = Co, Ni, Mn, etc.) cathode material possesses a typical hexagonal α-NaFeO_2_ layered structure, which belongs to the R$\bar{3}$m space group. The typical layered structure for LiMO_2_ is alternately arranged by two different layers, one is composed of the edge-sharing LiO_6_ octahedra, and the other one is formed by MO_6_ octahedra (Figure 4a). The Li-ions and M-ions are located at 3a and 3b sites of the octahedral structure respectively, and oxygen ions are located at 6c sites [41,42]. Owing to the similar ion size of Li^+^ (0.76 Å) and Ni^2+^ (0.69 Å) ions [43], Ni^2+^ are likely to occupy the Li^+^ sites, which is a disordered arrangement phenomenon called cation mixing [44], then R$\bar{3}$m structure will transform into the Fm$\bar{3}$m structure (Figure 4b). In the state of deep charging, the material shows structural instability because more lithium-ions are extracted from the host structure, which further leads to the migration of transition metal ions from the transition-metal layer to the lithium layer, finally resulting in more serious cation mixing (Figure 4c,d). The severe cation mixing will hinder the rapid extraction and insertion of lithium-ions during charge and discharge processes, which greatly affects the rate performance of Ni-rich cathode. The cation mixing phenomenon exists not only in the charging and discharging processes but also in the preparation process. In the synthesis process of cathode material, due to the existence of oxygen vacancy, the energy barrier for the Ni^2+^ migration to the lithium layer is small [45], leading to a more serious disordered arrangement. Therefore, in order to inhibit cation mixing, the calcination of Ni-rich cathode material should be carried out in an oxygen atmosphere. Choosing the appropriate ratio of lithium salt and sintering mechanism can also effectively inhibit cation mixing during calcination.

Figure 4e shows the typical curve between the differential capacity of Li/NCM811 battery and the battery voltage [46]. The NCM811 undergoes a phase transition from hexagonal (H1) to monoclinic (M) and then to hexagonal (H2 and H3) during the voltage increasing process [47,48,49]. When the voltage is above 4.11 V, the NCM811 electrode transforms from H2 to H3 phase, accompanied by the oxidation of lattice oxygen, and the cell volume changes dramatically, resulting in the cracking of the secondary particle. Therefore, the capacitance region corresponding to the phase transition from H2 to H3 is considered to be unstable, which is detrimental to maintaining reversible storage capacity. As shown in Figure 4f, when cycling to the same upper cutoff voltage, higher nickel content cathode materials experience larger c-axis lattice collapse [50]. Theoretical and experimental studies show that the lattice collapse of Ni-rich NCMs is related to the c-axis lattice shrinkage, during phase transformation from H2 to H3 [48,49].

Liu et al. [51] also revealed a common four-stage structural transition in Ni-rich cathodes by using high throughput operando neutron diffraction. In particular, they found that the Ni-rich cathodes had a universal structural evolution during about 75% delithiation, regardless of the nickel content or the substituent content. This evolution is hallmarked by the anomalous increase of the average TM–O bond lengths, which contradicts the traditional view that the TM–O bond lengths decrease in the charging process. This anomaly is caused by the direct oxidation of lattice oxygen ions, which is associated with the drastic decrease of oxygen-to-TM charge transfer gap. The finding reveals the elusive degradation mechanism of Ni-rich cathode. It shows that when charged to high SOC, structural evolution caused by oxygen redox might be the universal driving force for the capacity degradation of Ni-rich cathodes, which also provides valuable clues for stable cycling of layered oxide cathodes through stabilizing oxidized oxygen ions.

### 2.4. Microcracks

**Figure 5:**
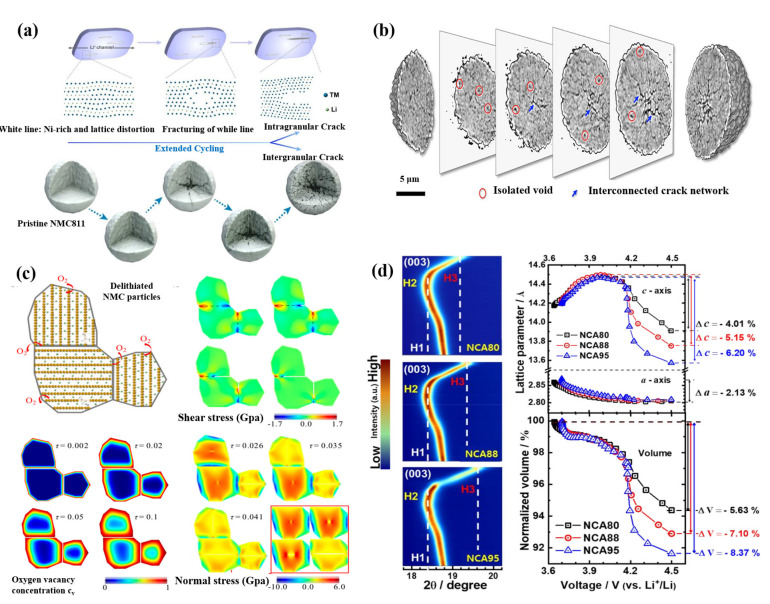
(**a**) Microstructure characteristics of intragranular and intergranular cracks. Reproduced with permission from ref. [53]. Copyright 2020 Elsevier. (**b**) 3D chemical mapping of a cycled (21 cycles) and fully charged NCA particle. Reproduced with permission from ref. [56]. Copyright 2018 American Chemical Society. (**c**) The relationship between oxygen vacancies and cracks of NCM. Reproduced with permission from ref. [59]. Copyright 2018 American Chemical Society. (**d**) The contour plots of (003) Bragg reflection of the NCA80, NCA88, and NCA95 cathodes in situ XRD measurements during the first charge process and variations of a-− and c-−axis lattice parameters and normalized unit cell volume as a function of the cell voltage. Reproduced with permission from ref. [54]. Copyright 2019 American Chemical Society.

It is generally considered that the mechanical failure of Ni-rich cathode materials will seriously affect the electrochemical performance of the battery, which is manifested by the initiation and development of cracks in the micro particles of the cathode materials. Surface reconstruction, lattice oxygen loss, phase transition and other factors may lead to particle cracks [52]. According to the location of the crack, it can be divided into intragranular and intergranular cracks, as shown in Figure 5a [53].

Researchers generally believe that H2→H3 phase transformation at high cut-off voltage is the main cause of intergranular microcracks in Ni-rich cathode materials. The effects of phase transformation mainly include anisotropic changes and sudden changes of lattice parameters [52,54]. Intergranular cracks make the contact failure between the primary particles, which greatly reduces the electrical conductivity inside the secondary particles, and further leads to heterogeneous SOC of the secondary particles. The relationship between SOC and porosity comes from charge compensation. Based on the charge compensation mechanism, during the charging process, unstable Ni^4+^ is reduced to Ni^2+^, while O^2−^ is oxidized to escape from the lattice for keeping the charge neutral. The escape of a large amount of lattice oxygen will generate oxygen vacancies, then the cathode materials release gases and generate pores. The interaction between porosity and SOC is a vicious circle. The formation of pores hinders the lithium diffusion path, resulting in the decrease of electrochemical activity between primary particles, and finally leading to the decrease of SOC. Uneven SOC also produces a large strain owing to the phase mismatch, which is the reason for the further expansion of cracks (Figure 5b–d) [55,56,57,58]. Mu et al. [59] studied the relationship between oxygen vacancy distribution and stress field in cathode particles by using finite element analysis (FEM), as shown in Figure 5c. With the increase of cycles, the oxygen vacancy increases. The shell, which is rich in oxygen vacancies, is more likely to expand than the core, which does not have vacancies, resulting in compressive stress near the surface and tensile stress, which is the main type stress-producing cracks, near the core. Sun et al. [54] reported that the capacity fading of Ni-rich cathode materials showed a strong correlation with the anisotropic volume variations, induced by the phase transition from H2 to H3, and the resulting extent of microcracking. As shown in Figure 5d, initial expansion and subsequent rapid shrinkage above 4.1V along the direction of c-axis are shown from the (003) reflection in contour plots, indicated by the dotted lines. The degree of unit cell shrinkage along the c-axis depends on the nickel content. Varying with the nickel content in the cathode, the c-axis lattice parameters of the three cathodes first increase to about 2.2%, and finally decrease to different values. The reductions of unit cell volumes for NCA80, NCA88 and NCA95 are 5.63%, 7.10% and 8.37%, respectively (Figure 5d). Consistent with the abrupt unit cell contraction along the c-direction, as the Ni fraction increases, the phase transformation from H2 to H3 is accelerated. On the other hand, the a-axis lattice parameter variation △*a* of cathode materials with different nickel contents is estimated by (110) reflection, which is the same as ~2.13% and is consistent with the results obtained by in situ XRD of Ni-rich NCMs [21]. As a result, the sudden phase transformation prevents the mechanical strain from dissipating in time and results in local strain concentration and severe microcracking [21].

In general, the influence mechanism of microcracks on the performance degradation of Ni-rich cathode materials can be understood as follows: in the pristine secondary particle of Ni-rich cathode materials, the charge transport and diffusion can occur without any obstacles, and the shortest geometrically optimal pathways can be taken. Inside the secondary particle with intergranular cracks, the liquid electrolyte has the ability to permeate through the microcracks, but the electrons have to bypass the microcracks. Therefore, the lithium-ions can diffuse through microcracks filled with electrolyte, and the electrons have to detour along the microcracks on account of the barriers caused by the intergranular cracks. Thus, the battery will suffer irreversible capacity loss and a significant increase in impedance [56].

## 3. Modifications

In order to deal with the above challenges of Ni-rich cathode materials under high-voltage operation, the researchers have proposed many modification methods. In the following parts, we will introduce in detail the modification strategies for Ni-rich cathode materials at high voltage from the aspects of element doping, surface coating, single-crystal fabrication, structural design, and multifunctional electrolyte additives.

### 3.1. Doping

#### 3.1.1. Single Element

Recently, element doping has also been widely used to control the internal microstructure of secondary particles, interface structure of the particle surface and primary particle size of cathode materials to optimize electrochemical properties. The commonly used cation doping elements for Ni-rich cathode materials are Na, Mg, Ca, B, Al, Ti, Zr and so on [60,61,62,63,64,65,66,67,68,69,70,71]. Some cation dopants can be incorporated into the crystal lattice interlayers of Ni-rich cathode materials as “pillar ions” to stabilize the bulk structure. It has been verified as an effective way for the reinforcement of electrochemical properties of high-voltage Ni-rich cathode materials.

**Figure 6:**
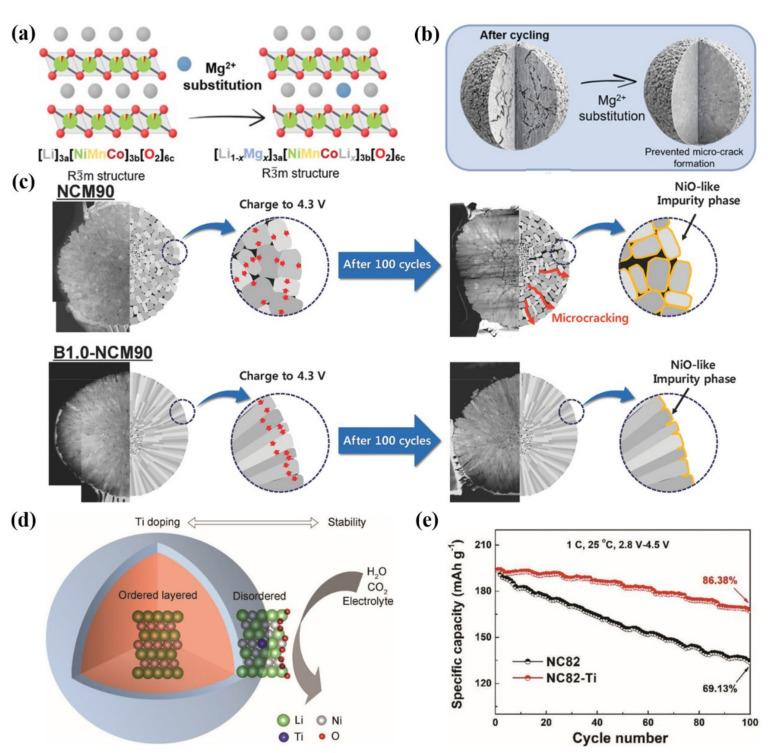
(**a**) The layered crystal structure of a Mg-substituted [Li_1−x_Mg_x_]_3a_[NiMnCoLi_x_]_3b_[O_2_]_6c_ structure with R$\bar{3}$m space group symmetry. (**b**) Schematic diagram of Ni-rich NCM without doping and Mg-substituted Ni-rich NCM after cycling. Reproduced with permission from ref. [69]. Copyright 2022 John Wiley and Sons. (**c**) Schematic diagram for the effect of boron-doping on the NCM90 cathode’s mechanical stability during charge and discharge cycling. Reproduced with permission from ref. [60]. Copyright 2018 John Wiley and Sons. (**d**) Schematic diagram for the mechanistic understanding of Ti doping. (**e**) The cycling performance for NC82 and NC82–Ti at 1 C in 2.8–4.5 V. Reproduced with permission from ref. [70]. Copyright 2019 John Wiley and Sons.

Schmuch et al. [69] indicated that 2 mol % Mg-doped cathode material possessed superior electrochemical properties. Its key electrochemical performance index is equivalent to that of commercial NCM811 material, and it has the additional advantage of reducing the Co content as a key raw material. The Mg^2+^ ions occupy the lithium layer and serve as “pillar ions” (Figure 6a), which can expand the lithium-ion channels, improve the reversibility of the structure and reduce the anisotropic lattice mismatch during the cycling, thus significantly enhancing the electrochemical stability and thermal stability of the electrode materials. As can be seen in Figure 6b, by the substitution of Mg, materials are less prone to generate microcracks, which is due to the suppressed and lower anisotropic volume change. In addition to Mg doping, appropriate content of electrochemically inactive doping elements such as Na and Al doping are also deemed as an effective means to alleviate structural collapse caused by deep de-lithiation of Ni-rich cathode materials during high voltage charging.

Sun et al. [60] used a boron-doping strategy to make anisotropic grains in the microstructure of Ni-rich cathode materials regularly arranged along with specific directions, so as to stabilize the internal microstructure of secondary particles, reduce the generation of cracks and avoid the chemical degradation caused by electrolyte erosion into the particles (Figure 6c). It has been confirmed by theoretical calculation that the doped boron ions could modify the surface energies. Therefore, this modification strategy contributes to achieving a highly textured microstructure, which can alleviate the intrinsic internal strain generated during deep charging. This finding suggests that extending the cycle life of Ni-rich cathodes through an optimal microstructure is considerable.

Pan et al. [70] reported that Ti-gradient doping could help to construct a disordered layered phase at the particle surface, which could greatly increase the robustness of the oxygen framework so that it could inhibit the corrosion of H_2_O, CO_2_ and electrolytes (Figure 6d). According to the first-principles calculation, the disordered layered phase caused by Ti-gradient doping has a low lithium-ion diffusion barrier, which contributes to high-rate performance. In addition, excellent cycling stability is realized due to the high structural stability (Figure 6e). This method makes full use of Ti doping to cause appropriate Li/Ni-exchange to form a disordered layered phase structure, and the design of the interface structure also provides a new idea for the design of other cathode materials.

Recently, Sun et al. [72] found that the introduction of a high-valence dopant can realize the primary particle size refinement, improving the cycling stability of the Ni-rich cathode materials. Among the many doped elements, Mo-doping has the best grain refining effect. Mo^6+^ can pin the grain boundaries, inhibit the growth of primary particles and confine the size to a submicrometer scale, thus greatly improving the cycling stability of the Ni-rich cathode materials. In the future, the influence of other high oxidation state dopants on the properties of Ni-rich cathode materials should be explored.

Some anions have larger electronegativity than O, therefore, it is usually reported that O^2−^ is replaced by the anions such as F^−^ and Cl^−^ to strengthen the binding energy between the cations and anions, which makes the structure more stable. Furthermore, due to the HF produced by the decomposition of liquid electrolytes, the surface of cathode materials is eroded. Due to the strong bond energy between the doped anions and metal ions, the dissolution of active substances in the cathode material can be effectively inhibited, especially in the electrolyte containing a large amount of electrolyte decomposition HF [73,74]. It is beneficial to improve the structural stability of the cathode material and the cycle life of the battery. Kim et al. [75] prepared F-doped Ni-rich NCMs (LiNi_0.8_Co_0.1_Mn_0.1_O_2−x_F_x_) with different amounts of F as a dopant. In comparison to the undoped NCM sample, the F-doped cathode materials show better cycling performance and rate capability because of the structural stability. However, the F-doped NCM that contained more than the optimal amount of F shows lower electrochemistry performance, due to the poor lithium-ion migration kinetics caused by the Li/Ni antisite defects [76]. Hence, controlling the appropriate doping content is key to achieving good electrochemical performance.

#### 3.1.2. Mixed Elements

**Figure 7:**
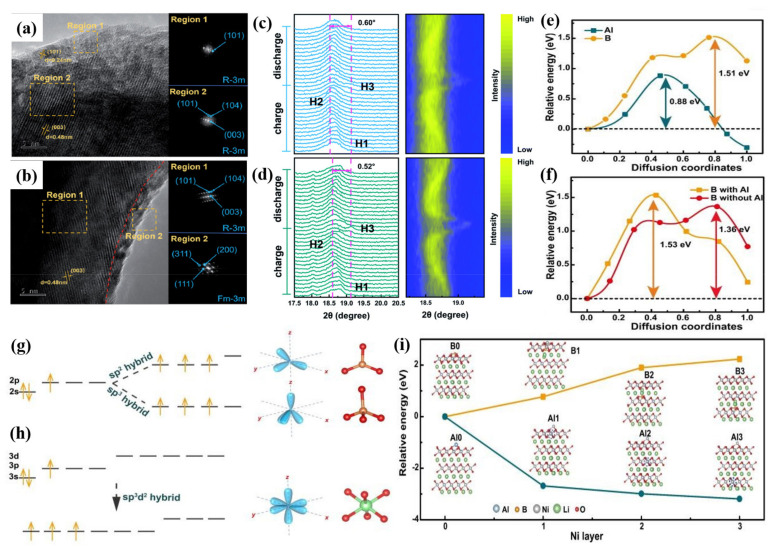
HRTEM images and corresponding FFT patterns for (**a**) NCM and (**b**) 0.5Ti@0.5F–NCM. In situ XRD tests of (**c**) NCM and (**d**) 0.5Ti@0.5F–NCM, including the detailed diffraction patterns and the corresponding contour plots. Reproduced with permission from ref. [77]. Copyright 2021 The Royal Society of Chemistry. (**e**) Al and B migration energy barrier from the first layer to the second layer. (**f**) B migration energy barrier from the surface to the first layer with Al and without Al. Orbit hybridization of (**g**) B and (**h**) Al. (**i**) The corresponding energy of different structures with doping Al and B to different layers. Reproduced with permission from ref. [78]. Copyright 2022 John Wiley and Sons.

The doped cation can maintain structural integrity and inhibit the formation of microcracks in Ni-rich cathode materials. F anion doping effectively enhances the binding force between transition metals and anions and effectively inhibits the precipitation and dissolution of active metals [73,74]. In view of the above two factors, co-doping of cation and anion can combine their advantages to improve the stability of intrinsic structure and interface and provide a synergistic effect for improving the electrochemical characteristic. Li et al. [77] designed Ti and F co-doped LiNi_0.8_Co_0.1_Mn_0.1_O_2_ cathode material and prepared it using a solid-state reaction. Co-doping of Ti and F contributes to the formation of an ultra-thin rock-salt phase on the cathode surface which serves as a protective layer for the Ni-rich cathode surface (Figure 7a,b) so that the electrochemical properties of the material are greatly improved. Co-doping of Ti and F can also inhibit the H2–H3 phase transition of the Ni-rich cathode materials in charge/discharge processes (Figure 7c,d). The optimal Ti^4+^ and F^−^ co-doped sample 0.5Ti@0.5F-NCM shows a superior discharge capacity retention much higher than NCM at 1 C under the high temperature (45 °C). Ti-doping contributes to structural stability and F-doping helps to achieve thermal stability. The results show that co-doping is a feasible modification strategy to enhance the electrochemical performance of Ni-rich cathodes for LIBs.

Guo et al. [78] synthesized Al/B co-doped Ni-rich cathode materials (Li(Ni_0.88_Co_0.09_Mn_0.03_)_0.985_Al_0.005_B_0.01_O_2_, NCM-AB) via solid-state reaction. B dopant has a tendency to form sp^2^ or sp^3^ orbital hybridization with the lattice oxygen in NCM (Figure 7g). It shows a triangular configuration (BO_3_) or tetrahedral configuration (BO_4_) which are different from the TMO_6_ octahedron configuration in NCM. Since the radius of B ions is much smaller than that of TM cations, the doping of B at the interstitial position will lead to lattice distortion that hinders the diffusion of lithium ions in the bulk. On the other hand, the 3s and 3p orbitals of Al could hybridize with the d orbital, and the sp^3^d^2^ orbital hybridization coordinates with the lattice oxygen to form AlO_6_ octahedron, which is similar to octahedron TMO_6_ (Figure 7h). Density functional theory calculations show that Al preferentially binds to oxygen and the diffusion barrier of Al is much lower than that of B (Figure 7e,f). When Al preferentially occupies the sites, it prevents the massive diffusion of B in the bulk phase. The energy of Al on the surface is much higher than that in the inside layers. In contrast, B is stable on the surface and possesses much lower energy than that of the inside layers (Figure 7i). Therefore, the formation of B-rich surface and Al-rich bulk contributes to the cooperative stabilization of the bulk structure and surface chemical stability of cathode materials. NCM-AB shows excellent capacity retention during the cycling process. Precisely controlling dopant site and depth in the cathode material contributes to inhibition of the adverse bulk phase transformation and surface reconstruction during the high-voltage cycling. This work shows that co-doping is a rational approach for developing high-performance electrode materials, which could also help to carry out related studies about new co-dopant chemistry.

Overall, element doping is an efficient way for the enhancement of Ni-rich cathode materials’ electrochemical properties. Nevertheless, when the doping content of inactive elements exceeds the appropriate amount, the capacity of the material will be greatly reduced. Therefore, controlling the appropriate doping content is the key to achieving excellent electrochemical performance. Nowadays many elements have been reported to serve as dopants for Ni-rich cathode materials but how their electrochemical performance changes with the doped elements remain to be verified. From the perspective of commercialization, the cost of the dopants and doping methods are also worth considering. Therefore, in order to further develop high-voltage Ni-rich cathode materials, it is necessary to conduct more basic research on the doping effect and economical doping methods.

### 3.2. Surface Coating

Surface coating has been considered to be a simple and effective method to protect the electrode surface. It refers to coating a protective layer on the cathode materials surface to inhibit or weaken the serious side reactions at the interface between cathode electrode and electrolyte, so as to reduce the dissolution of the transition metal and enhance the surface structure stability, eventually improving the electrochemical performance of cathode materials. Various coating substances have been explored, such as metal oxides, fluorides and phosphates, conductive polymers, and fast-ion conductors, etc.

The physical protection coating materials such as Al_2_O_3_ [79], CeO_2_ [80], ZrO_2_ [81], TiO_2_ [82], SiO_2_ [83], CaO [84], ZnO [85], Y_2_O_3_ [86], etc, have been commonly used to isolate the contact between the cathode electrode and the electrolyte, effectively preventing HF corrosion and heightening the electrochemical characteristics of the cathode materials. Metal fluorides (LiF [87], AlF_3_ [88], etc) and phosphates (AlPO_4_ [89], FePO_4_ [90], Zn_3_(PO_4_)_2_ [91], etc) are also used for coating the surface of the cathode materials because of their stable physicochemical properties. However, these substances have poor ionic conductivity and electronic conductivity, which will add to the interface impedance of the material and decrease the dynamic performance of the battery. Therefore, conductive polymers with outstanding ionic conductivity and/or electronic conductivity (PANI-PEG [92], PEDOT [93], PPy [94], PAN [95], etc) and fast-ion conductors with good ionic conductivity (Li_2_SnO_3_ [96], Li_3_PO_4_ [84,97,98], Li_4_Ti_5_O_12_ [99], LiTa_2_PO_8_ [100], etc) have been gradually applied to coat Ni-rich cathode materials; these modified materials have shown excellent electrochemical properties.

**Figure 8:**
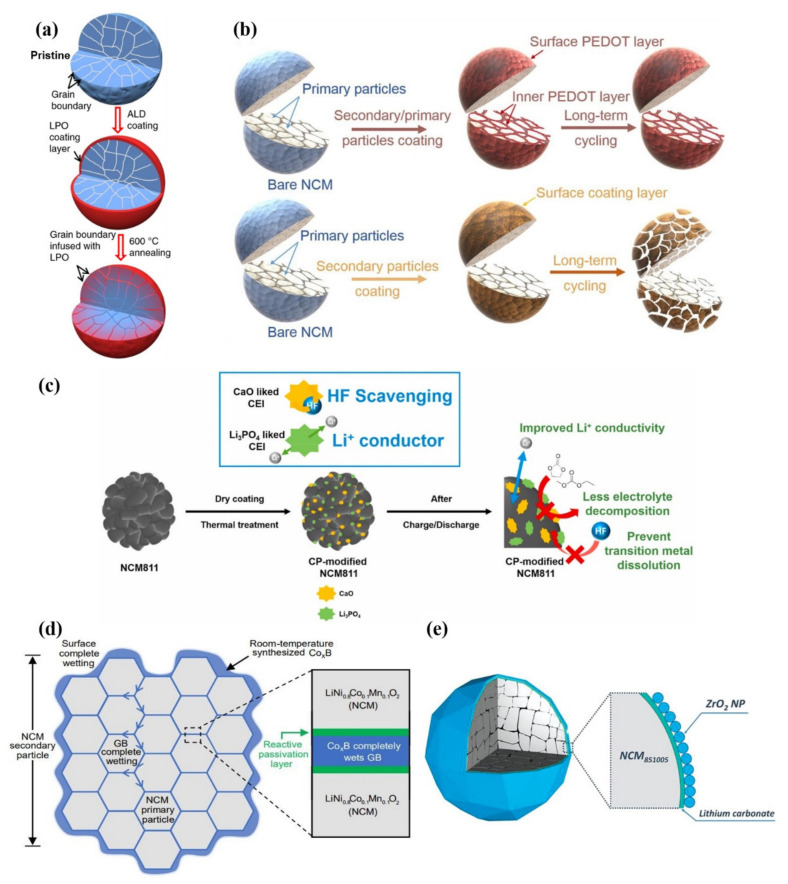
(**a**) Schematic diagram for the evolution of the LPO coating layer on the secondary particle. Reproduced with permission from ref. [98]. Copyright 2018 Springer Nature. (**b**) Grain boundary modification can ensure the structural stability of the cathode better than simple coating. Reproduced with permission from ref. [93]. Copyright 2019 Springer Nature. (**c**) Role of the surface-modified NCM811 cathode material dually functionalized by Ca_3_(PO_4_)_2_. Reproduced with permission from ref. [84]. Copyright 2021 Elsevier. (**d**) Schematic “coating–plus–infusion” microstructure with Co_x_B for NCM primary particles. Reproduced with permission from ref. [102]. Copyright 2021 Springer Nature. (**e**) Schematic coating morphology. The protective coating has a unique bilayer structure, consisting of ZrO_2_ NPs and (mostly) lithium carbonate. Reproduced with permission from ref. [81]. Copyright 2022 John Wiley and Sons.

Zhang et al. [98] proposed an effective method to improve the structural and interfacial stability of cathode secondary particles. Coating and infusing the Li_3_PO_4_ (LPO) solid-state electrolyte along the grain boundaries of secondary particles can help to stabilize the structure and interface (Figure 8a) and contribute to the long-term cycling stability of the battery. The improvement for performance is related to the modification of grain boundaries by solid electrolyte, which can promote the rapid transport of lithium-ions, avoid the penetration of liquid electrolyte and the destruction of surface structure by liquid electrolyte, so that it can achieve the purpose of reducing cracks.

Cao et al. [92] reported that upon combining polyaniline (PANI) and polyethylene glycol (PEG), the conductive polymers possessed outstanding electronic conductivity and excellent ionic conductivity, which contributed to the modification of LiNi_0.8_Co_0.1_Mn_0.1_O_2_ cathode material surface. In order to reduce the corrosion of HF acid in the electrolyte, 3,4-ethylene dioxythiophene (PEDOT), an ultra-conformal conductive polymer, has been used to react with the electrolyte [93,101]. As shown in Figure 8b, the surface of secondary and primary particles in the NCM cathodes are built with an ultra-conformal protective skin, which could greatly strengthen the crystal and interfacial structural stability. On the contrary, traditional surface coating only on the secondary particles could not prevent the primary particles from the corrosion of the electrolyte. The electrolyte could still penetrate along the grain boundaries, thus leading to the structural degradation and further attenuation of capacity and voltage after long-term cycling [93].

As shown in Figure 8c, Yim et al. [84] fabricated a composite protective layer composed of CaO and Li_3_PO_4_ at the cathode surface of NCM811, which was derived from Ca_3_(PO_4_)_2_ precursor by dry coating technology. CaO is used as HF scavenging material to prevent corrosion between acid and cathode. In this way, the dissolution of transition metal ions by HF could be inhibited. Additionally, Li_3_PO_4_ possesses excellent ionic conductivity, which can provide quick lithium-ion transport channels. The combination of CaO and Li_3_PO_4_ contributes to the excellent cycling stability and rate capability of the NCM811 cathodes. This work shows that the functional CEI layer formed by Ca_3_(PO_4_)_2_ precursor is considered a deal modification measure for enhancing the electrochemical properties of Ni-rich cathode materials.

Cho et al. [102] reported a simple room-temperature synthesis route to improve Ni-rich cathode materials. At room temperature, with the driving force for interface reaction, nanoscale Co_x_B metallic glass is totally wrapped on the surface of secondary particles and injected into grain boundaries (GBs) between primary particles (Figure 8d). Co_x_B possesses good mechanical properties and is not easy to cut or break at the nanometer scale. Co_x_B has a strong reactivity with the surface oxygen of the cathode material which can stabilize the surface oxygen. Therefore, this design helps electrode material to achieve superior mechanical and electrochemical properties. After 500 cycles at 1 C in the voltage range of 2.8–4.3 V, the pouch-type full battery still has 95.0% capacity retention. This work is different from the typical surface coating. The coating and injection modification strategy can significantly improve electrochemical performance. It is also a common modification strategy for state-of-the-art electrodes by injecting oxides with transition-metal boride/silicide/phosphide metallic glasses to form “functional cermets”.

Ma et al. [81] proposed a wet-coating method to improve the Ni-rich cathode materials. A nano-scale coating consisting of ZrO_2_ non-agglomerated nanoparticles (NPs) monolayer and Li_2_CO_3_ layer were successfully constructed on the surface of the secondary particles (Figure 8e). The uniform coating inhibits the interfacial reaction between the electrolyte and the cathode material, and it can limit gas production at the same time. This work shows that it is an effective coating strategy for improving the cycling stability of Ni-rich cathode materials.

### 3.3. Single-Crystal Fabrication

The morphology of traditional Ni-rich cathodes is a polycrystalline spherical secondary particle that is agglomerated by primary particles. The primary particles are randomly gathered, which leads to the strong grain boundary stress because of the anisotropy of lithium-ion diffusion. Furthermore, the increase in boundary stress will also result in the electron transfer failure among primary particles and accelerate the erosion of the electrolyte. Recently, fabricating single-crystal cathode particles has been considered as an effective strategy to reduce the grain boundary stress [103,104]. Single-crystal cathode materials possess high crystallinity and isotropy orientation so that the requirements of advanced mechanical strength and more homogeneous electrochemical reactions can be realized.

**Figure 9:**
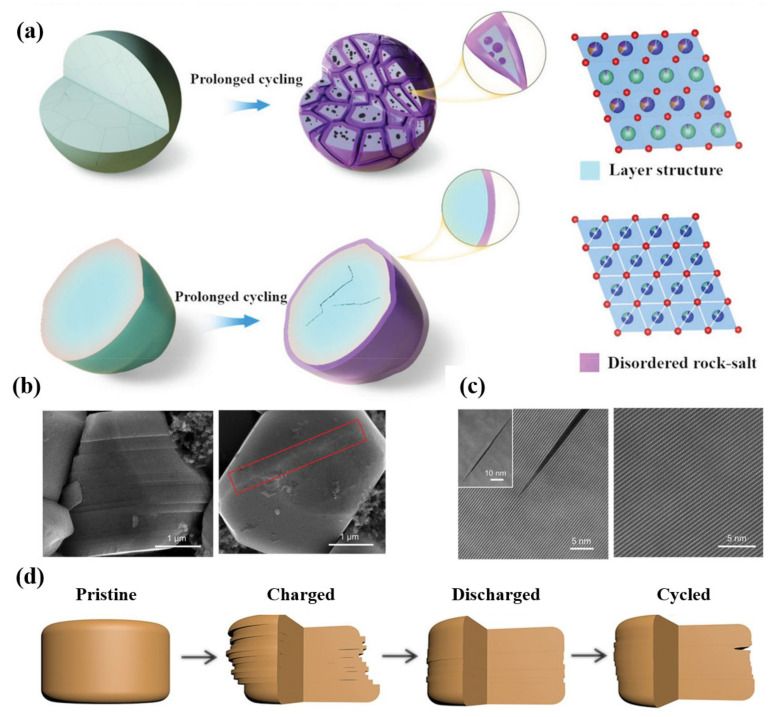
(**a**) PC–NCM622 particles and SC–NCM622 particles after long–term cycling. Blue spheres represent transition metal ions, green spheres represent lithium ions, and red spheres represent oxygen ions. Reproduced with permission from ref. [105]. Copyright 2022 John Wiley and Sons. (**b**) SEM image of single-crystalline NCM76 initially charged to 4.8 V and discharged to 2.7 V (after being charged to 4.8 V). (**c**) STEM images of single-crystalline NCM76 at charge status and discharge status (cycled in a full cell between 2.7 and 4.4 V for 120 cycles). (**d**) Schematic diagram for the structural evolution of single-crystalline NCM76 during cycling. Reproduced with permission from ref. [107]. Copyright 2020 The American Association for the Advancement of Science.

Zhao et al. [105] compared the electrochemical properties of single crystal LiNi_0.60_Co_0.20_Mn_0.20_O_2_ (SC-NCM622) and polycrystalline LiNi_0.60_Co_0.20_Mn_0.20_O_2_ (PC-NCM622) cathode materials in pouch cells. After the prolonged cycling, the cracks of SC-NCM622 are less numerous, but for PC-NCM622, more intergranular cracks were formed. Compared with PC-NCM622 particles, only a few parts of SC-NCM622 particles change from a layered structure (R$\bar{3}$m) to a disordered rock-salt phase (Fm$\bar{3}$m), these phase transformations are only distributed on the surface of SC-NCM622 particles (Figure 9a). The uniform phase inside the SC-NCM622 particle makes it maintain the integrity of the structure. The SC-NCM622 particles have better cycling stability than PC-NCM622 particles, especially at higher voltage of cycling. The study suggests that it is a promising way of developing LIBs with high-energy and cycling stability by controlling the single-crystal structure of particles.

Although the single-crystal cathodes can avoid intergranular fractures, it is still inevitable to generate intragranular cracks, which is also the main degradation mechanism of the single-crystal cathodes [106]. Different from the uncontrollability of intragranular in polycrystalline particles, cracks along (003) direction in single-crystal particles keep stable during the cycling process. Bi et al. [107] observed reversible planar slips and microcracks along (003) the crystal plane in a single-crystal Ni-rich cathode. When discharged to 2.7 V (after being charged to 4.8 V), most of the glided layers inside the single crystals glide back to their original positions and the microcracks disappear; the original structure has been maintained (Figure 9b,c). Nevertheless, the microcracks will be generated after repeated gliding near the crystal surface, and then the electrolyte will destroy the new surface (Figure 9d). This work demonstrates that the reversible formation of microstructure defects is related to the local stress caused by Li concentration change in the lattice. Therefore, reducing the crystal size to below a certain size (3.5 μm), changing the structural symmetry to absorb the accumulated strain energy and optimizing the charging depth without sacrificing much reversible capacity can effectively stabilize the single-crystal Ni-rich cathode materials.

Recently, Xiao et al. [108] demonstrated that gas generated from single-crystal NCM was much less than that of polycrystalline NCM at a high voltage operation. Compared with polycrystals, gas generation from single-crystal cathode materials required a higher electrochemical driving force. The significant reduction of boundaries and surface area in single-crystal NCM inhibits severe surface side reactions which can also reduce gas release. This study shows that utilizing single-crystal materials is an effective pathway to improve battery safety from a material perspective.

### 3.4. Structural Design 

When nickel content of the layered oxide cathode material is higher than 80% during the cycling process, the interface between cathode and electrolyte becomes instable and sudden structural collapse occurs as well, eventually resulting in performance attenuation of the battery. In order to obtain high capacity and high structural stability, researchers have carried out many attempts to design a concentration gradient for Ni-rich cathode materials, including core-shell (CS) [109], core-shell concentration gradient (CSG) [110,111], full concentration gradient (FCG) [112] and two-slopes full concentration gradient (TSFCG) [113] which are used to improve structural stability, reduce side reactions and adverse volume change.

Sun et al. [109] proposed the core-shell structure of Ni-rich cathode materials which could help to realize the high capacity and thermal stability of cathode materials. The high capacity is delivered from the LiNi_0.8_Co_0.1_Mn_0.1_O_2_ core, and the excellent thermal stability stems from the LiNi_0.5_Mn_0.5_O_2_ shell. However, the study shows that due to the obvious difference between the core material and shell composition, the degree of shrinkage of the core and shell region is different, which may lead to the gradual separation of the core and shell, hindering the transfer of electrons and the effective diffusion of lithium-ions. This discontinuity induces serious deterioration of battery performance.

**Figure 10:**
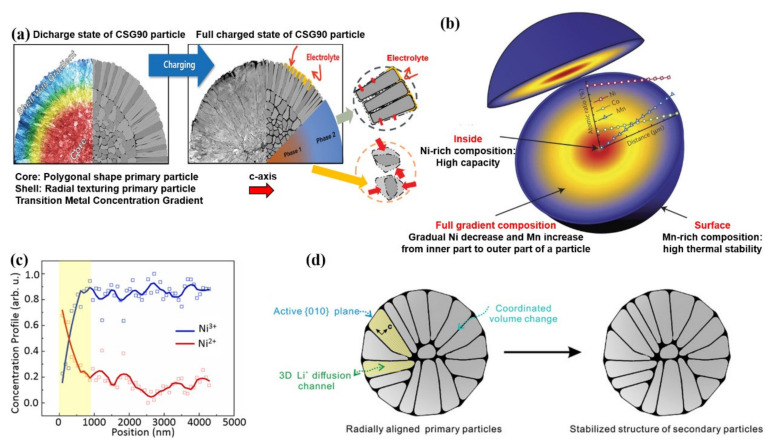
(**a**) Schematic illustration for CSG90 and CC90 cathodes at discharge and charge state showing the internal morphological difference and the sustained damage. Reproduced with permission from ref. [111]. Copyright 2019 John Wiley and Sons. (**b**) Schematic illustration for the FCG lithium transition-metal oxide particle. Reproduced with permission from ref. [112]. Copyright 2012 Springer Nature. (**c**) The Ni^2+^ and Ni^3+^ concentrations from surface to the center regions. Reproduced with permission from ref. [114]. Copyright 2021 Springer Nature. (**d**) Schematic illustration for the relationship between stress distribution and particle internal structure. Reproduced with permission from ref. [115]. Copyright 2019 John Wiley and Sons.

Furthermore, Sun et al. [110] proposed a core-shell structure with a concentration gradient. Slightly different from the core-shell structure, the element concentration gradient exists in part of the shell. The concentration of Ni decreases towards the surface, while the concentration of Mn and Co increases correspondingly. The outer layer of the concentration-gradient and the surface composition of LiNi_0.46_Co_0.23_Mn_0.31_O_2_ help to realize the structural stability and thermal stability of the material. Sun et al. [111] synthesized a multi-compositional particulate LiNi_0.9_Co_0.05_Mn_0.05_O_2_ cathode (CSG90), in which LiNi_0.94_Co_0.038_Mn_0.022_O_2_ is surrounded by a 1.5 μm thick concentration gradient shell at the center of the particle. The composition for the particle surface is LiNi_0.841_Co_0.077_Mn_0.082_O_2_. The stable crystallographic texture and radial primary particles can disperse the force generated by the sudden adverse phase transition during charging and prevent the electrolyte from penetrating into the bulk of the particle (Figure 10a). Compared with the CSG90, there is more NiO rock-salt phase generated on the particle surface for conventional cathodes (CC90).

Based on previous studies, Sun et al. [112] further invented a Ni-rich cathode material with a novel structure, in which the concentration of Ni decreases linearly from the center to the outer layer of the particle, while the concentration of Mn increases gradually (Figure 10b). This design enables the material to remain structurally stable during the high-voltage cycling process. In order to maximize the Ni content in the inner part of the particle and Mn content near the particle surface, Sun et al. [113] designed a two-slope full concentration gradient structure to further improve the electrochemical performance of cathode materials. The concentration changes of Ni and Mn show two linear regions with different slopes in the central layer and the shell layer. The concentration of Ni decreases slowly in the central region and decreases rapidly in the shell layer. The concentration of Mn increases slowly in the central region and increases rapidly near the surface region. Such chemical distribution of components generates high capacity and thermal stability. Each TSFCG secondary particle is composed of rod-like primary particles. The rod-like primary particles are radially arranged from the center of the particle, providing outstanding cycling stability and rate capability owing to the unique microstructure of the TSFCG secondary particle.

In order to separate the valence gradient effect of Ni and understand its fundamental stabilization mechanism, Yang et al. [114] designed and synthesized a novel LiNi_0.8_Co_0.1_Mn_0.1_O_2_ (VG-NCM811) material with uniform composition and a hierarchical valence gradient structure. As shown in Figure 10c, the concentration of Ni^2+^ is high in the near-surface region and decreases rapidly in the near-surface 900 nm range. Finally, the concentration of Ni^2+^ gradually decreases toward the center, while the concentration of Ni^3+^ rapidly increases and then slowly increases from the surface to the center. Compared with the conventional materials, the Ni valence gradient materials have better cycling performance. The study demonstrates that VG-NCM811 possesses a better structural stability and thermal stability as well, which can be ascribed to the Ni-valence gradient in the subsurface section of the secondary particles.

As shown in Figure 10d, Sun et al. [115] synthesized a micrometer-sized Ni-rich LiNi_0.8_Co_0.1_Mn_0.1_O_2_ secondary cathode material which was made up of radially oriented single-crystal primary particles. Combining the exposed active surface with the 3D lithium-ion diffusion channels from the surface to the center, the diffusion coefficient of lithium-ions is significantly increased. In addition, the primary particles with consistent crystal orientation can significantly coordinate volume change caused by expansion/contraction so that it can alleviate the inter-crystalline stress and inhibit the pulverization of secondary particles. Owing to the compatible crystal orientation and 3D Li^+^ diffusion, the rate capability and cycling stability of the single-crystal NCM811 cathode materials are greatly improved. This work provides an effective method for enhancing the performance of Ni-rich cathode materials through anisotropic morphology modulation.

### 3.5. Multifunctional Electrolyte Additives

CEI and SEI films are mainly composed of varieties of organic compounds generated by the decomposition reactions of electrolyte solvents. Therefore, the use of multifunctional electrolyte additives is viewed as a general strategy for stabilizing CEI and SEI films of Ni-rich cathode-based LIBs [116,117,118]. Electrolyte additives are often used to attenuate side reactions between electrolyte and electrode and prolong service life at high voltages. Electrolyte additives have higher HOMO energy levels and lower LUMO energy levels than conventional electrolyte solvents such as EC, DMC and DEC [119,120,121]. Therefore, prior to the decomposition reaction of the electrolyte solvents, the electrolyte additive will be oxidized on the cathode surface and reduced on the anode surface to form uniform CEI and SEI films [122]. In addition to their film-forming capability, multifunctional additives have a high affinity for HF and PF_5_, which can alleviate HF-driven degradation, such as the dissolution of transition metal ions and gas release. 

**Figure 11:**
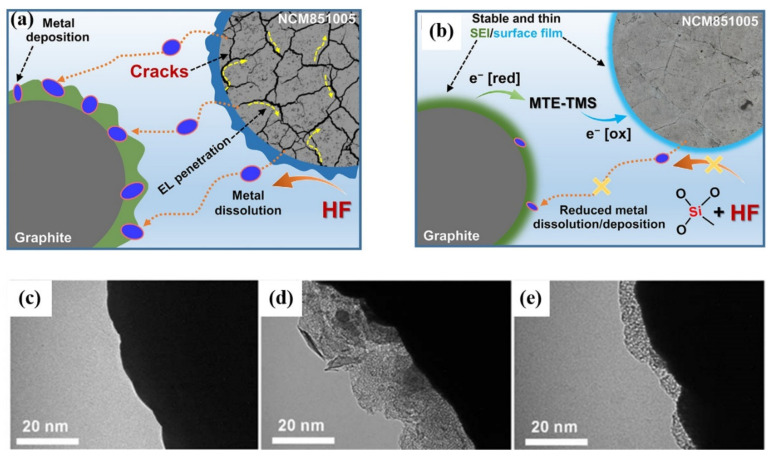
Schematic diagram for interfacial phenomena occurring in the graphite‖NCM851005 full-cells, cycled between 3.0 and 4.25 V in the standard electrolyte (**a**) without and (**b**) with MTE-TMS additive. Reproduced with permission from ref. [123]. Copyright 2020 Elsevier. Corresponding TEM images (**c**) Pristine NCM851005 cathode and cathodes cycled (**d**) without and (**e**) with AEDB additive, taken from graphite‖NCM851005 full-cells after 100 cycles. Reproduced with permission from ref. [11]. Copyright 2021 John Wiley and Sons.

In recent years, researchers have proposed several types of multifunctional additives in order to effectively form protective films on both cathode and anode surfaces. Trabesinger et al. [123] proposed methoxytriethyleneoxypropyltrimethoxysilane (MTE-TMS) as a novel multifunctional electrolyte additive for the stabilization of Ni-rich layered LiNi_0.85_Co_0.1_Mn_0.05_O_2_ (NCM851005) cathode‖graphite anode interface. The surface of Ni-rich cathode materials cycled with standard electrolyte is covered with thick CEI film. On the contrary, with an MTE-TMS additive, a thin surface film is formed at the interface between the cathode and electrolyte, which is composed of organic compounds and MTE-TMS decomposition products. Si-containing species in MTE-TMS decomposition products contribute to the surface structural robustness so that it could inhibit the generation of cracks and significantly reduce the dissolution of transition metal ions (Figure 11a,b). Based on this mechanism, the MTE-TMS additive causes an improvement in cycling performance and coulombic efficiency. Pham et al. [11] reported 2-aminoethyl diphenyl borate (AEDB) as a multifunctional electrolyte additive for improving the electrochemical properties of the battery. AEDB possesses a special chemical structure for relieving surface degradation reactions. The B–O bond helps to eliminate reactive species such as HF and PF_5_. The NH_2_ group contributes to forming a protective surface film on the cathode. The surface of Ni-rich cathode materials cycled with standard electrolyte is rich in inorganic species and metal fluorides which derive from LiPF_6_. In contrast, with AEDB additive, the surface is composed of organic species and decomposition products of AEDB, and the thin surface film is rather uniform and smooth (Figure 11c–e). Since the cathode surface is covered with a protective film, the side reactions between cathode and electrolyte would be inhibited, reducing the degradation of the cathode surface and the generation of microcracks. Therefore, the battery using AEDB as an electrolyte additive possesses a capacity retention of 88% after 100 cycles at the high cutoff voltage of 4.35 V, under the same conditions, the capacity retention of the battery using standard electrolyte is only 21%. This work shows it is a simple strategy for stabilizing the surface and bulk of both Ni-rich cathodes at high-voltage operation. Recently, Hu et al. [124] found that lithium difluorophosphate (LiDFP) is a serviceable electrolyte additive for maintaining the cyclic stability of Ni-rich cathode materials at an ultra-high voltage (4.8 V). With 1% LiDFP, the polycrystalline LiNi_0.76_Co_0.10_Mn_0.14_O_2_ (NCM76) cathode upcycling 200 cycles possesses an impressive capacity retention of 97% in 2.8–4.8 V. The excellent cycling stability is attributed to the stable interface formed by the decomposition of LiDFP, which contributes to the rapid transmission of lithium-ions at the interface. This work not only effectively inhibits the dissolution of transition metals and the surface reconstruction of cathodes, but also facilitates the uniform distribution of lithium in bulk particles, thus alleviating strain and inhibiting the formation of cracks.

## 4. Conclusions and Perspectives

The increasing demand for power LIBs with high energy density, long-time cycle life and safety stability requires cathode materials to have excellent performance under high voltage operation. Ni-rich layered oxide cathode materials possess a high capacity and have become the principal candidates for long-range EVs in recent years.

In this review, the main problems existing in Ni-rich cathode materials under high-voltage operation and the recent progress of modification methods are discussed. The challenges for Ni-rich cathode materials mainly include the stability of electrode interface, gas release, phase transformation, microcracks, etc. Some strategies to address these challenges are put forward, including element doping, surface coating, single-crystal fabrication, structural design and use of electrolyte additives. Element doping is a simple modification method, which can stabilize the bulk structure and regulate the internal microstructure of secondary particles, interface structure of the particle surface and primary particle size of cathode materials. Nevertheless, when the doping content of inactive elements exceeds the appropriate amount, the capacity of the material will be greatly reduced. Therefore, controlling the appropriate doping content is the key to achieving good electrochemical performance. Surface coating has been considered an effective method to inhibit or weaken serious side reactions and enhance surface structure stability, thus it helps to improve the electrochemical performance of cathode materials. Single-crystal fabrication contributes to realizing the mechanical stability of the material and inhibiting the generation of cracks, thus alleviating side reactions between cathode materials and the electrolytes during the high-voltage cycling, reducing the release of gases to improve battery safety. Multifunctional electrolyte additives can alleviate the HF-driven degradation and can be oxidized on the cathode to form a stable CEI film, thus effectively reducing the severe side reactions under high voltage.

Overall, researchers have proposed many modification strategies to improve the Ni-rich cathode materials under high voltage operation. There remain still many challenges to overcome, and it is still a tough road until the commercial application of high-voltage cathode materials. An in-depth understanding of materials by advanced analysis techniques can help to improve the capacity and cycle stability of materials. In addition, the internal mechanism of doping and coating strategies, and the synthesis of Ni-rich cathode materials for single-crystalline or concentration gradient need to be deeply analyzed. Furthermore, studies are needed to combine the synergistic effect of the bulk/surface modification strategies with particle engineering. Therefore, it is believed that with the continuous development of Ni-rich cathode materials, it will play an important role in the large-scale utilization of LIBs in the future.

## Data Availability

Data sharing not applicable. No new data were created or analyzed in this study. Data sharing is not applicable to this article.
